# Longitudinal Changes in Visual Field and Optical Coherence Tomography Parameters in Patients with Pituitary Microadenomas: A 12-Month Controlled Study

**DOI:** 10.3390/cancers18213563

**Published:** 2026-11-06

**Authors:** Monika Sarnat-Kucharczyk, Małgorzata A. Janik, Paweł Janik, Beata Kos-Kudła, Ewa Mrukwa-Kominek

**Affiliations:** 1 Department of Ophthalmology, Faculty of Medical Sciences in Katowice, Medical University of Silesia, 40-752 Katowice, Poland; emrowka@poczta.onet.pl; 2 Kornel Gibinski University Clinical Centre, Medical University of Silesia, 40-514 Katowice, Poland; 3 Institute of Biomedical Engineering, Faculty of Science and Technology, University of Silesia in Katowice, 41-205 Sosnowiec, Poland; malgorzata.janik@us.edu.pl (M.A.J.); pawel.janik@us.edu.pl (P.J.); 4 Department of Endocrinology and Neuroendocrine Tumors, Medical University of Silesia, 40-514 Katowice, Poland; beatakos@ka.onet.pl

**Keywords:** pituitary microadenoma, visual field, optical coherence tomography, GC-IPL, RNFL, mean deviation, kinetic perimetry

## Abstract

Background/Objectives: The ophthalmological course of pituitary microadenomas without overt chiasmal compression remains poorly understood. This study evaluated visual field and retinal structural changes over 12 months in patients with conservatively managed pituitary microadenomas. Methods: This prospective controlled study included 23 patients with pituitary microadenomas and 23 age- and sex-matched healthy controls. Examinations performed at baseline and after 12 months included best-corrected visual acuity, static and kinetic perimetry, and optical coherence tomography assessment of the ganglion cell–inner plexiform layer (GC-IPL), peripapillary retinal nerve fibre layer (RNFL), and central macular thickness. Results: Visual acuity and visual field index remained stable in both groups. Mean deviation was lower in the microadenoma group in both eyes at baseline and after 12 months. Baseline mean deviation differentiated patients from controls, with an area under the curve of 0.820 for the right eye and 0.773 for the left eye. Pattern standard deviation increased in both eyes of patients with microadenomas but remained unchanged in controls. Kinetic perimetry showed no consistent pattern of longitudinal change. GC-IPL, RNFL, and central macular thickness remained stable during follow-up. Conclusions: Conservatively managed pituitary microadenomas may be associated with static perimetric differences despite preserved visual acuity and stable retinal structure. The clinical significance of these exploratory findings remains uncertain and warrants evaluation in larger confirmatory studies with longer follow-up.

## Simple Summary

Pituitary microadenomas are small tumors that usually do not compress the optic pathways or cause obvious visual problems. However, it remains unclear whether they may be associated with subtle changes in visual function or retinal structure over time. In this study, patients with pituitary microadenomas and healthy individuals underwent visual field testing and optical coherence tomography at baseline and after 12 months. Visual acuity, overall visual field function, and retinal layer thickness remained stable during follow-up. Nevertheless, patients with microadenomas showed slightly reduced visual field sensitivity, while some visual field parameters changed over time. These functional findings were not accompanied by retinal thinning. The results suggest that conservatively managed pituitary microadenomas are not associated with clinically apparent visual deterioration over 12 months. Larger studies with longer follow-up are needed to determine whether the subtle visual field differences have clinical importance.

## 1. Introduction

Pituitary neuroendocrine tumors (PitNETs), historically termed pituitary adenomas, are among the most common sellar lesions. PitNETs constitute a heterogeneous group of tumors that differ in hormonal activity, growth pattern, and biological behaviour [1,2,3,4]. Owing to the close anatomical relationship between the pituitary gland, optic chiasm, and anterior visual pathways, tumor growth beyond the confines of the sella may result in compressive optic neuropathy [5,6]. Visual field defects, reduced visual acuity, impaired color vision, and retinal structural alterations associated with compressive macro-PitNETs are well established [7,8,9].

Compression of the optic chiasm predominantly affects the decussating axons originating from the nasal hemiretinae, typically producing temporal visual field loss [10]. Although bitemporal hemianopia is regarded as the classic presentation, early defects may be subtle, asymmetric, or limited to the superior temporal quadrants. Moreover, structural injury to retinal ganglion cells and their axons may develop before clinically apparent deterioration in visual acuity or before characteristic visual field defects become detectable using conventional perimetry [11,12].

Micro-PitNETs, conventionally defined as tumors measuring less than 10 mm in maximum diameter, generally remain confined to the sella and do not cause radiologically evident compression of the optic chiasm. Most incidentally detected pituitary microlesions are therefore managed conservatively, with clinical, hormonal, and radiological follow-up determined by their size, functional status, and proximity to the optic chiasm [13,14]. Consequently, routine neuro-ophthalmic surveillance is primarily focused on patients with larger tumors or lesions abutting the anterior visual pathways [15,16,17]. Nevertheless, subtle functional or retinal structural abnormalities have occasionally been reported in patients without overt chiasmal compression [18]. Whether these findings represent early or subclinical visual pathway involvement, interindividual anatomical variability, effects associated with hormonal disturbances, or nonspecific measurement variability remains uncertain [19].

Automated static perimetry, kinetic perimetry, and optical coherence tomography (OCT) enable detailed assessment of visual function and retinal neural structures. In particular, measurements of peripapillary retinal nerve fiber layer (RNFL) and macular ganglion cell–inner plexiform layer (GC-IPL) thickness may reveal structural abnormalities not detectable during routine ophthalmic examination [20,21,22]. However, most available observations in patients with micro-PitNETs are cross-sectional, and it remains unclear whether any subtle functional or structural differences persist or progress over time.

The aim of this controlled longitudinal study was therefore to evaluate changes in visual field parameters and retinal structure over a 12-month period in patients with micro-PitNETs compared with healthy controls. Kinetic and standard automated perimetry were combined with OCT assessment of the macular GC-IPL and peripapillary RNFL to determine whether patients with micro-PitNETs demonstrate measurable functional or structural progression despite the absence of overt chiasmal compression.

## 2. Materials and Methods

### 2.1. Study Design and Participants

This prospective longitudinal controlled study included 23 patients diagnosed with pituitary microadenoma and 23 control participants. Participants underwent comprehensive ophthalmological assessment at baseline and after a 12-month follow-up period.

Patients in the microadenoma group were recruited from those admitted to the Endocrinology Department.

The study group consisted of patients with a confirmed diagnosis of pituitary microadenoma. All pituitary microadenomas were clinically non-functioning, as established by endocrinological assessment. The control group consisted of individuals without pituitary pathology, selected as a reference population for comparison of functional and structural ophthalmological parameters.

All ophthalmological examinations were repeated after 12 months. The primary longitudinal analysis assessed within-group changes between baseline and 12 months in patients with pituitary microadenomas and controls. Changes over time were subsequently compared between the two groups to determine whether the evolution of functional or structural parameters differed between patients with microadenomas and healthy controls.

### 2.2. Inclusion and Exclusion Criteria

The inclusion criteria common to both groups were age ≥ 18 years, written informed consent, intraocular pressure ≤ 20 mmHg, and best-corrected distance visual acuity (BCVA) ≥ 0.1 in decimal notation (Snellen equivalent, 5/50). An additional inclusion criterion for the study group was an established diagnosis of pituitary microadenoma confirmed by magnetic resonance imaging (MRI). Control participants had no history of diagnosed pituitary disease.

Participants were excluded if they had ocular media opacities involving the cornea, lens, or vitreous. Retinal exclusion criteria included retinal degeneration, macular degeneration, diabetic retinopathy, retinal detachment, and previous retinal detachment surgery. Other ocular exclusion criteria were post-traumatic ocular changes, nystagmus, glaucoma, previous refractive surgery, and ocular surgery within the preceding six months. Participants with high myopia, defined as a spherical equivalent below −6.00 diopters, or hyperopia exceeding +6.00 diopters were also excluded. Further exclusion criteria were psychiatric disorders, systemic diseases that could prevent completion of the planned examinations, pregnancy, and breastfeeding.

### 2.3. Ophthalmological Examination

All participants underwent a comprehensive ophthalmological examination at baseline and at the 12-month follow-up visit. All participants underwent slit-lamp biomicroscopy and fundus examination, including clinical assessment of the optic disc. The examination protocol included visual acuity assessment, evaluation of visual field function using kinetic and standard automated perimetry, and structural assessment of the posterior segment of the eye using optical coherence tomography.

#### 2.3.1. Visual Acuity

BCVA was assessed using a Snellen chart and recorded in decimal notation. For statistical analysis, decimal visual acuity values were converted to logarithm of the minimum angle of resolution (logMAR) units.

#### 2.3.2. Static Automated Perimetry

Static visual field testing was performed using the Humphrey Field Analyzer, Carl Zeiss Meditec, Dublin, CA, USA. The 24-2/30-2 testing strategy with the SITA Fast algorithm was used. Global visual field indices, including visual field index (VFI), mean deviation (MD) and pattern standard deviation (PSD), were recorded and analyzed. Visual field examinations meeting predefined reliability criteria were included in the analysis. Only reliable visual fields were included, defined according to standard reliability criteria (fixation losses, false-positive and false-negative responses within acceptable limits).

#### 2.3.3. Kinetic Perimetry

Kinetic visual field examination was performed using kinetic perimetry (Goldmann perimeter, Haag-Streit Inc., Bern, Switzerland). Visual field boundaries were assessed using standardized kinetic stimuli Goldmann III4e. Kinetic perimetry examinations were considered reliable when fixation was maintained throughout the test and responses were reproducible upon repeated stimulus presentation. Changes in the extent and configuration of the visual field were evaluated longitudinally and compared between patients with pituitary microadenomas and control participants.

#### 2.3.4. Optical Coherence Tomography

Structural assessment of the retina and optic nerve was performed using the CIRRUS 6000 spectral-domain optical coherence tomography system (Carl Zeiss Meditec AG, Jena, Germany). The OCT examination included measurements of the peripapillary retinal nerve fiber layer (RNFL), macular ganglion cell–inner plexiform layer (GC–IPL), and macular thickness. Images were processed using the device’s automated segmentation software (version 11.5.2.545332). GC–IPL thickness was assessed as an average value and within six sectors: superior, superonasal, inferonasal, inferior, inferotemporal, and superotemporal. The RNFL was evaluated as an average thickness and separately within the superior, nasal, inferior, and temporal quadrants. Macular thickness was assessed using the central thickness value. Only scans of adequate quality and without clinically relevant segmentation artifacts were included in the analysis.

### 2.4. Statistical Analysis

Statistical analyses were performed using Statistica version 13 (TIBCO Software Inc., Palo Alto, CA, USA). The distribution of continuous variables was evaluated using the Shapiro–Wilk test and inspected visually with histograms. Continuous data are reported as mean and standard deviation (SD) for variables with an approximately normal distribution or as median and interquartile range (IQR) for variables that did not meet the assumption of normality. Categorical variables are presented as numbers and percentages.

Baseline demographic and clinical characteristics were compared between patients with pituitary microadenomas and healthy controls. Continuous variables were compared between the two independent groups using the unpaired Student’s *t*-test when the relevant assumptions were satisfied and the Mann–Whitney U test otherwise.

Within-group changes between baseline and the 12-month examination were assessed using the paired Student’s *t*-test for normally distributed differences and the Wilcoxon signed-rank test for non-normally distributed differences. Changes over time were additionally calculated as the difference between the 12-month and baseline measurements (Δ = value at 12 months − value at baseline) and compared between the study and control groups using the unpaired Student’s *t*-test or the Mann–Whitney U test, as appropriate. All tests were two-sided, and a *p*-value below 0.05 was considered statistically significant.

Right and left eyes were analyzed separately and were not pooled as independent observations. Within-eye changes between baseline and 12 months were assessed using paired tests.

For comparisons of continuous variables analyzed using *t*-tests, effect sizes were expressed as Cohen’s *d* and calculated using G*Power version 3.1.9.7 (Heinrich Heine University Düsseldorf, Düsseldorf, Germany; https://www.psychologie.hhu.de/arbeitsgruppen/allgemeine-psychologie-und-arbeitspsychologie/gpower; accessed on 25 May 2025) [23].

For nonparametric tests, the effect size was expressed as *r_g_* = *Z*/(*N*^0.5^), where Z denotes the Wilcoxon test statistic. Effect sizes were interpreted as small (*d* = 0.20 and *r_g_* = 0.1), medium (*d* = 0.50 and *r_g_* = 0.3), or large (*d* = 0.80 and *r_g_* = 0.5). All tests were two-sided, and a *p*-value below 0.05 was considered statistically significant.

Given the exploratory nature of the analyses, no adjustment for multiple comparisons was applied, and all reported p-values are unadjusted. Accordingly, statistically significant findings should be interpreted as exploratory.

## 3. Results

### 3.1. Baseline Parameters

A total of 46 participants completed the 12-month follow-up and were included in the final analysis: 23 patients with pituitary microadenomas managed conservatively and 23 healthy controls. Baseline demographic and clinical characteristics are presented in Table 1.

Tumor dimensions for individual patients are presented in Supplementary Table S1.

### 3.2. Static Visual Field

#### 3.2.1. VFI

VFI values showed only minimal changes during the 12-month follow-up. In microadenoma group, mean VFI changed from 98.96% to 98.65% in the right eye and from 98.70% to 99.00% in the left eye. In control group, the corresponding values changed from 99.26% to 99.43% and from 99.22% to 99.39%, respectively. No significant baseline-to-12-month changes were observed in either microadenoma group (right eye, *p* = 0.201; left eye, *p* = 0.424) or control group (right eye, *p* = 0.262; left eye, *p* = 0.308). Furthermore, no statistically significant differences in VFI were found between the groups at baseline or at the 12-month follow-up (all *p* > 0.05).

#### 3.2.2. MD

**Figure 1:**
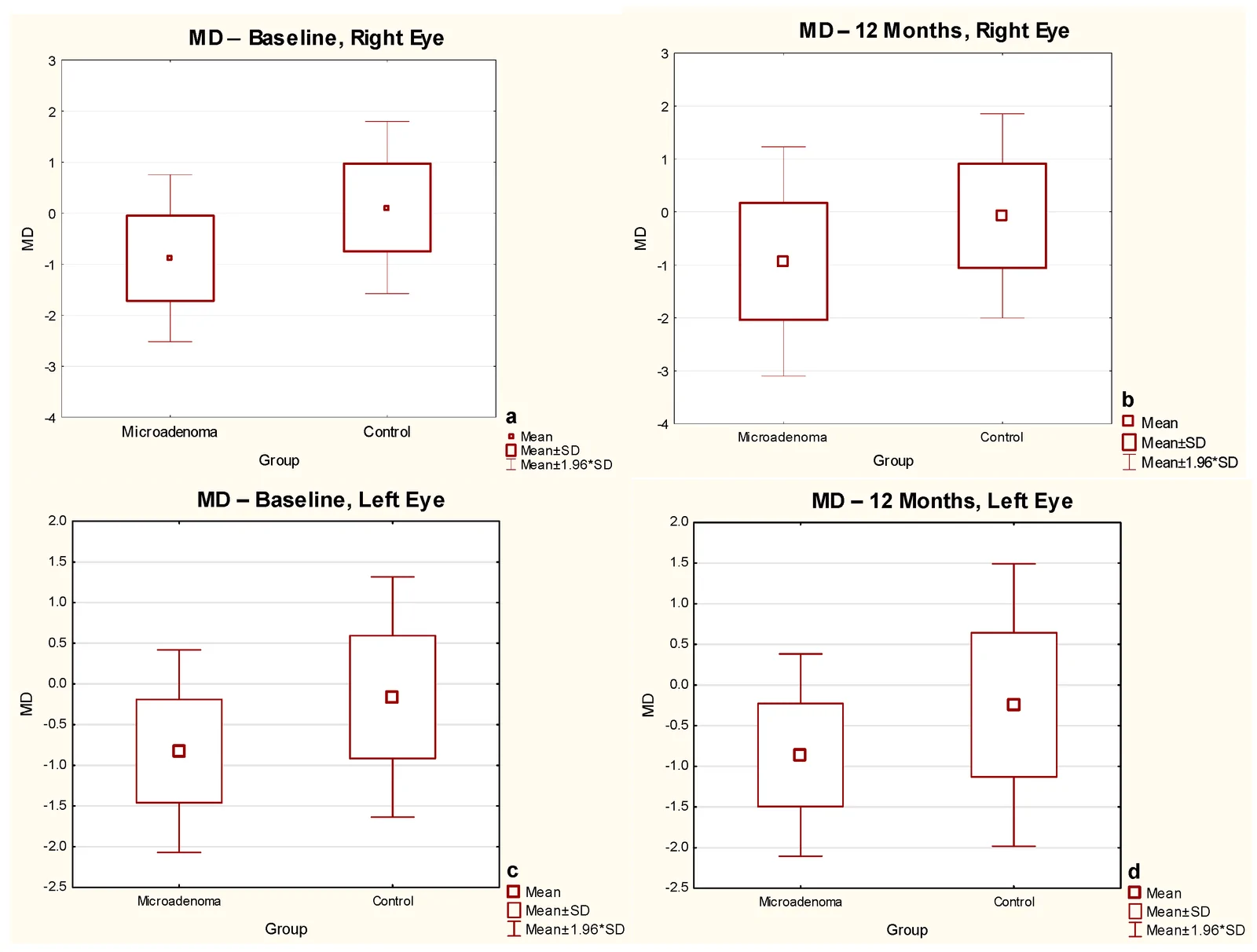
Comparison of visual field mean deviation (MD) between the microadenoma and control groups at baseline and after 12 months. (**a**) Right eye at baseline; (**b**) right eye at 12 months; (**c**) left eye at baseline; and (**d**) left eye at 12 months. Boxes represent the interquartile range, horizontal lines indicate medians, and whiskers represent the minimum and maximum values. The asterisk (*) denotes multiplication.

MD values differed significantly between microadenoma group and control group in both eyes at baseline (right eye: *p* < 0.001, effect size *r_g_* = 0.55 (large); left eye: *p* = 0.001, effect size *d* = 0.95 (large)) and at 12 months (right eye: *p* = 0.019, effect size *d* = 0.82 (large); left eye: *p* = 0.009, effect size *d* = 0.80 (large)). Among the analyzed static perimetry parameters, MD demonstrated consistent differences between the groups. The results obtained for the right and left eyes at baseline and after 12 months are presented in Figure 1.

**Figure 2:**
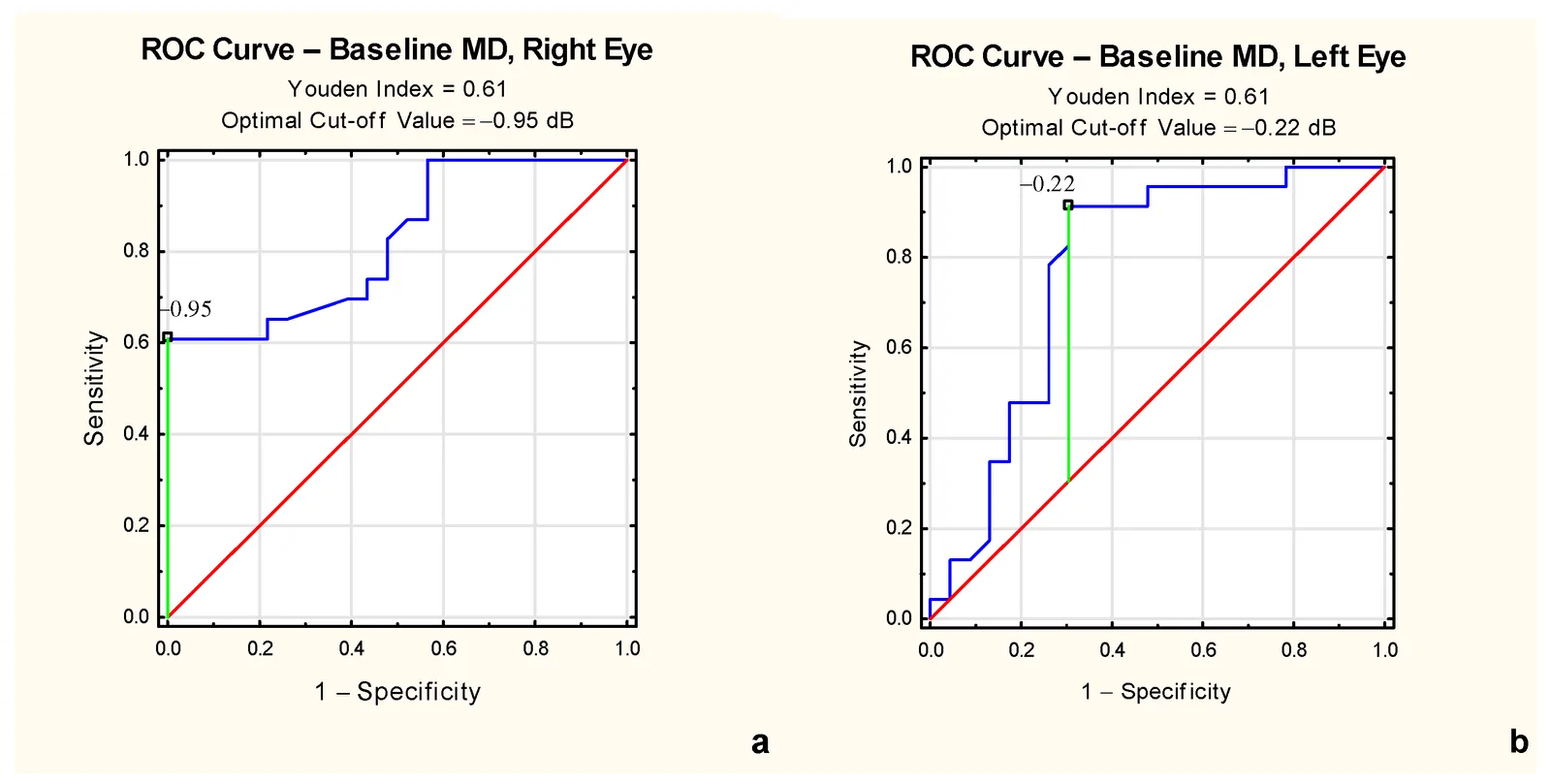
Receiver operating characteristic curves illustrating the ability of baseline visual field mean deviation to discriminate between the microadenoma and control groups: (**a**) right eye and (**b**) left eye. The red line represents the reference line, the blue line represents the ROC curve, and the green line indicates the selected cut-off value. AUC—area under the receiver operating characteristic curve; MD—mean deviation.

Given the significant between-group differences in baseline MD, ROC curve analysis was performed to assess its ability to distinguish patients with microadenomas from healthy controls. MD significantly discriminated between the microadenoma and control groups in both the right eye (AUC = 0.82 ± 0.061 (Lower 95% CI = 0.7, Upper 95% CI = 0.941), *p* < 0.001) and the left eye (AUC = 0.773 ± 0.074 (Lower 95% CI = 0.628, Upper 95% CI = 0.918), *p* < 0.001), as shown in Figure 2. Lower MD values were associated with the microadenoma group. At the selected cut-off values of −0.95 dB for the right eye and −0.22 dB for the left eye, sensitivity and specificity were 0.609 and 1.000, respectively, for the right eye and 0.913 and 0.696, respectively, for the left eye.

#### 3.2.3. PSD

**Figure 3:**
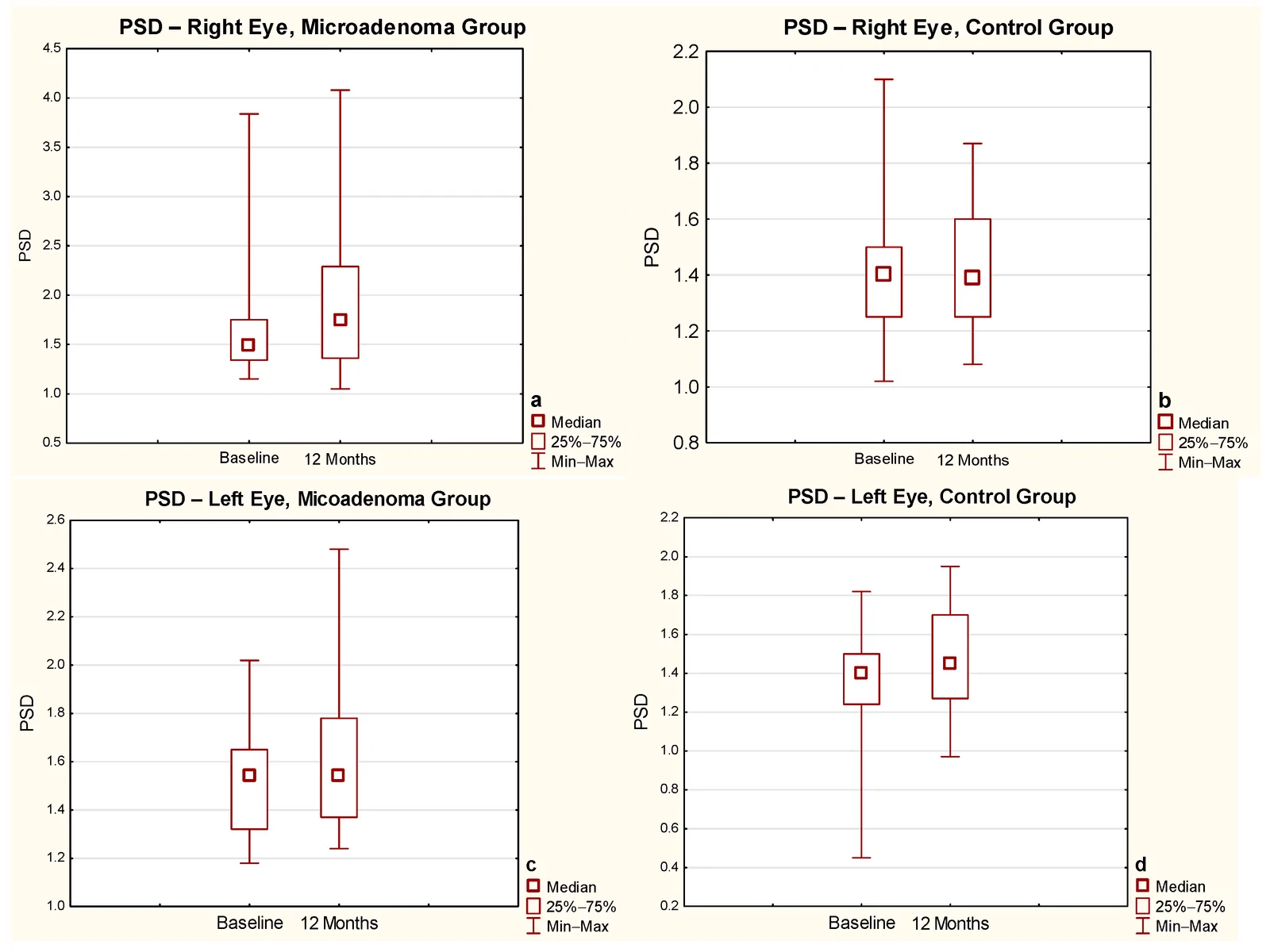
Pattern standard deviation (PSD) at baseline and after 12 months in the microadenoma and control groups: (**a**) right eye, microadenoma group; (**b**) right eye, control group; (**c**) left eye, microadenoma group; (**d**) left eye, control group. Squares indicate medians, boxes represent the interquartile range (25th–75th percentiles), and whiskers indicate the minimum and maximum values.

PSD changed significantly over the 12-month follow-up in the microadenoma group in both the right eye (1.65 to 1.90 dB, *p* = 0.012, effect size (*d* Cohen) = 0.57 (medium)) and the left eye (1.52 to 1.62 dB, *p* = 0.014, effect size *d* = 0.56 (medium)). No significant changes were observed in the control group (right eye, *p* = 0.748, effect size *d* = 0.07 (small); left eye, *p* = 0.072, effect size *d* = 0.39 (small)). The groups did not differ significantly at baseline. At 12 months, PSD was significantly higher in the microadenoma group than in the control group in the right eye (1.90 vs. 1.41 dB, *p* = 0.003, effect size *r_g_* = 0.44 (medium)), whereas no significant between-group difference was observed in the left eye. Changes in PSD from baseline to 12 months in the microadenoma and control groups are presented in Figure 3.

**Figure 4:**
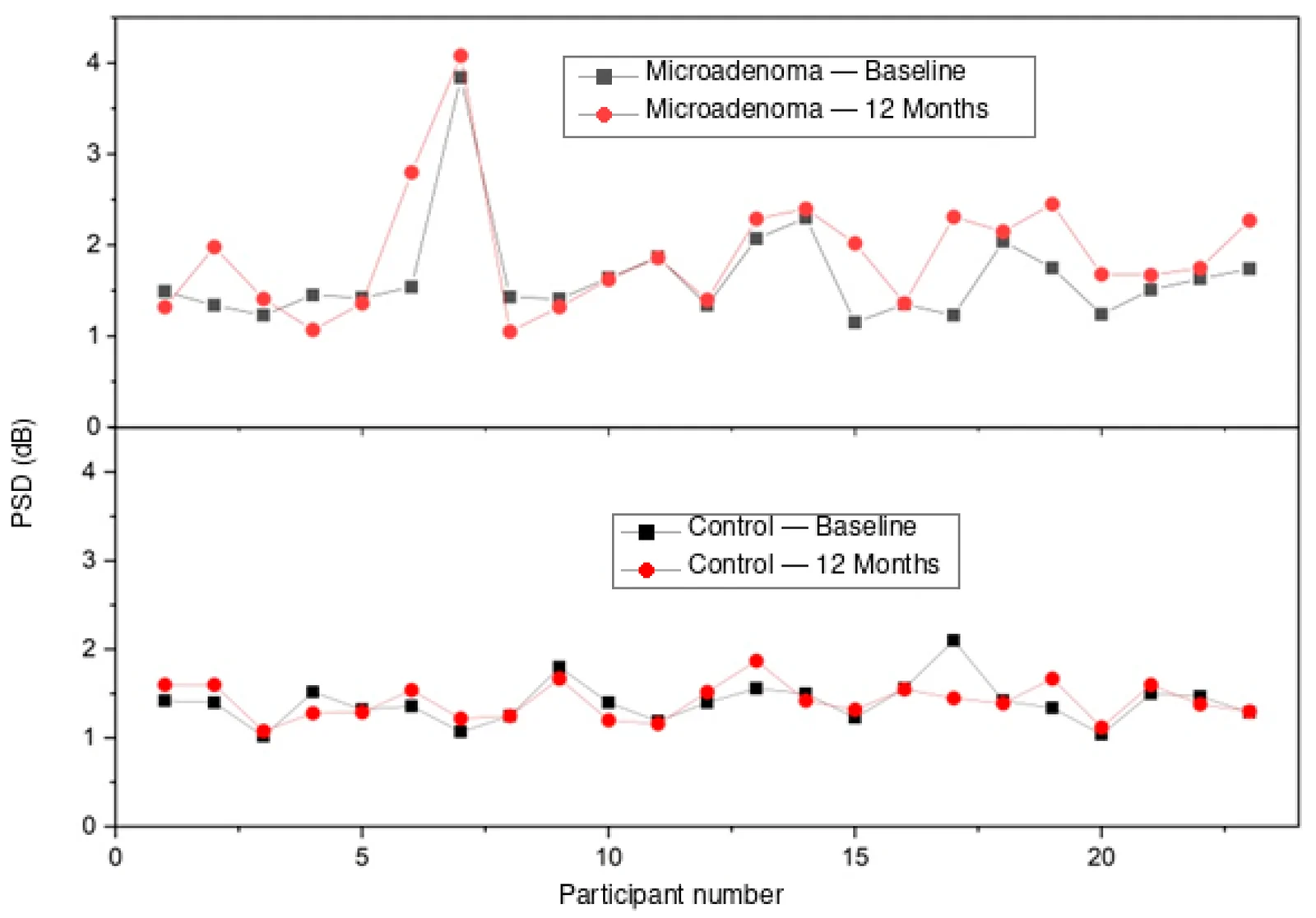
Individual participant PSD values at baseline and at the 12-month follow-up. Pattern standard deviation (PSD) values are shown for the microadenoma group (**upper panel**) and the control group (**lower panel**). Squares indicate baseline measurements, and red circles indicate measurements at the 12-month follow-up. Each position on the horizontal axis represents one participant, with paired measurements displayed at the same position. PSD values are expressed in decibels (dB).

Individual participants’ paired PSD values at baseline and at the 12-month follow-up are shown separately for the microadenoma and control groups in Figure 4.

### 3.3. Kinetic Visual Field

Kinetic perimetry showed broadly comparable results between the microadenoma and control groups. Most within-group longitudinal and between-group comparisons were not statistically significant. Isolated nominally significant differences were observed for selected meridians and eyes; however, these findings were inconsistent across directions and were not considered to represent a coherent pattern of visual-field change. Detailed results are presented in Supplementary Table S2.

### 3.4. OCT

OCT analysis included average and sectoral GC-IPL and peripapillary RNFL thickness, as well as macular thickness.

#### 3.4.1. GC-IPL

Longitudinal changes in average and sectoral GC-IPL thickness are summarized in Supplementary Table S3. No significant changes were observed in the microadenoma group over the 12-month follow-up. An isolated nominally significant change was found in the superior-temporal GC-IPL sector of the right eye in the control group (*p* = 0.044); however, no corresponding between-group difference was observed. All remaining longitudinal and between-group comparisons were not statistically significant.

#### 3.4.2. RNFL

Longitudinal changes in average and sectoral peripapillary RNFL thickness are summarized in Supplementary Table S4. No significant changes between baseline and the 12-month follow-up were observed in either the microadenoma or control group. At 12 months, superior RNFL thickness was significantly greater in the microadenoma group than in the control group in both the right (*p* = 0.020) and left eyes (*p* = 0.039). No corresponding differences were detected at baseline. All remaining between-group comparisons were not statistically significant.

#### 3.4.3. Macula

Macular thickness measurements at baseline and 12-month follow-up are summarized in Supplementary Table S5. No significant longitudinal changes were observed in either the microadenoma or control group. Furthermore, macular thickness did not differ significantly between the groups at either time point (all *p* > 0.05).

## 4. Discussion

The preservation of BCVA and the absence of significant longitudinal changes in VFI are consistent with the generally indolent short-term course reported for conservatively managed pituitary microadenomas. In a large multicentre cohort of 459 patients with non-functioning pituitary microadenomas, Hamblin et al. reported a probability of tumor growth of only 1.3% at 1 year, while visual field deterioration secondary to chiasmal compression was an exceptionally rare event during longer follow-up [24]. Similarly, a systematic review by Fernández-Balsells et al. demonstrated that worsening of visual field defects was uncommon among conservatively managed pituitary incidentalomas and non-functioning adenomas, with complications occurring predominantly in larger lesions [25]. Our findings extend these observations by providing standardized ophthalmological evidence that global visual function remains stable over 12 months in patients with microadenomas without overt chiasmal compression.

Despite preserved BCVA and near-normal VFI, MD was consistently lower in patients with pituitary microadenomas than in healthy controls. Lee et al. previously reported that greater pituitary macroadenoma volume was associated with lower MD and higher PSD values [26]. However, their study included patients with macroadenomas, often accompanied by visual field defects, whereas our patients had small lesions without overt chiasmal compression. Ogra et al. also showed that normal or near-normal visual acuity may coexist with visual field defects in patients with pituitary adenomas [27]. Thus, the lower MD observed in our patients may reflect subtle functional abnormalities that are not detectable by visual acuity or VFI. The mechanism underlying this finding remains unclear, particularly in the absence of radiological evidence of chiasmal compression. One possible interpretation is subtle visual pathway dysfunction without retinal structural changes detectable by OCT [28]. This hypothesis requires further investigation, as the present study was designed to assess longitudinal functional and structural changes rather than their underlying mechanisms.

MD and VFI are global indices and may not fully reflect the distribution of defects associated with pituitary lesions. Gesslein et al. found that these indices did not reliably characterize visual field defects in patients with compression of the visual pathways and emphasized the importance of evaluating individual test locations [29]. Therefore, the lower MD values observed in our study should not be interpreted as evidence of compressive optic neuropathy. Although baseline MD showed moderate-to-good discrimination between the groups in the ROC analysis, this result should be considered exploratory because of the small sample size and lack of external validation. Further studies combining detailed visual field analysis, OCT, and neuroimaging are needed to determine whether this finding has clinical relevance.

This cautious approach is consistent with the recommendation that evaluation of pituitary-related visual dysfunction should integrate quantitative indices with the spatial distribution of defects and, where appropriate, complementary peripheral field assessment [15].

The different longitudinal patterns of MD and PSD reflect the distinct aspects of the visual field captured by these indices. MD describes the overall deviation from age-adjusted normal sensitivity, whereas PSD reflects spatial irregularity. Thus, the observed increase in PSD despite stable MD suggests greater unevenness of visual field sensitivity without a significant longitudinal change in its overall deviation from age-adjusted norms.

The PSD increases were modest in absolute magnitude despite reaching statistical significance in both eyes and should be interpreted alongside the stable MD, VFI, and OCT measurements.

Kinetic perimetry revealed only isolated statistically significant differences, without a consistent pattern across eyes or meridians. Although kinetic testing complements central static perimetry by assessing the peripheral extent of the visual field, the abnormalities observed in our cohort did not form a reproducible pattern suggestive of chiasmal involvement. Given their inconsistency and the number of comparisons performed, these findings may reflect measurement variability or chance rather than clinically meaningful progression.

OCT measurements remained stable during the 12-month follow-up. No significant changes in average or sectoral GC-IPL thickness, average or quadrant-specific RNFL thickness, or central macular thickness were found in patients with microadenomas. Previous studies have reported inner retinal thinning in patients with pituitary tumours. Cennamo et al. found reduced ganglion cell complex (GCC) and peripapillary RNFL thickness in patients with pituitary macroadenomas without radiologically evident chiasmal compression [6]. Although GCC and GC-IPL are not identical measurements, both include the macular ganglion cell and inner plexiform layers; GCC also includes the macular RNFL.

Orman et al. reported lower macular ganglion cell, macular nerve fibre layer, and peripapillary RNFL measurements in patients with pituitary tumours despite normal visual fields [12]. Zhang et al. also suggested that GCC loss may occur before RNFL thinning [11]. However, these studies mainly included patients with larger tumours or heterogeneous sellar lesions and were cross-sectional. Their results may therefore not be directly applicable to patients with conservatively managed microadenomas.

In our patients, the lower MD values were not accompanied by GC-IPL or RNFL thinning. It remains unclear whether this represents an early functional abnormality preceding structural loss or simply reflects the variability of perimetric measurements. Similar observations were reported by Duseikaite et al. in a cohort including patients with both pituitary microadenomas and macroadenomas. Although temporal RNFL was thinner in the overall pituitary adenoma group than in healthy controls, inferior RNFL thickness was lower in patients with macroadenomas than in those with microadenomas [30]. This may indicate that structural retinal abnormalities are more readily detectable in larger tumours. In patients with chiasmal compression, Yum et al. found characteristic thinning of the nasal macular GC-IPL [31]. Menon et al. reported that RNFL thinning, particularly in the nasal and temporal quadrants, was associated with visual field loss and more advanced radiological involvement [32]. The greater superior RNFL thickness found in our microadenoma group at 12 months does not correspond to this typical pattern. It was not present at baseline and was not associated with a significant longitudinal change. Similarly, the isolated superior-temporal GC-IPL change in the control group had no corresponding between-group difference. These isolated findings are therefore more likely to reflect measurement variability or multiple testing than a structural effect of the microadenoma.

### 4.1. Study Limitations

Although the prospective design and inclusion of a matched healthy control group strengthen the study, the relatively small sample size and 12-month follow-up period may have limited the ability to detect subtle longitudinal changes and may restrict the generalizability of the findings. Therefore, the results should be interpreted as exploratory and confirmed in larger, preferably multicentre cohorts with longer follow-up. Given the large number of statistical comparisons and the relatively small sample size, isolated nominally significant findings should be interpreted cautiously and considered exploratory.

Axial length was not measured, so its potential influence on OCT measurements could not be assessed despite the exclusion of high refractive errors.

Right and left eyes were analyzed separately using paired longitudinal comparisons; however, a joint mixed-effects model incorporating both eyes and visits was not used.

The static perimetry analysis focused on global visual field indices (MD, PSD, and VFI). Pointwise visual field data were not retained for subsequent analysis; therefore, we could not assess the distribution of deficits across the four quadrants or determine whether the increase in PSD was associated with changes predominantly affecting a particular quadrant. This limits the anatomical interpretation of our findings and our ability to assess whether the observed changes followed a pattern suggestive of chiasmal involvement.

Although anatomical location and radiological progression were evaluated on MRI, their potential associations with visual function were not analyzed in this study.

The ROC analysis was exploratory and was performed in a relatively small cohort without external validation. Therefore, the identified discriminatory performance and cut-off values should not be interpreted as clinically validated diagnostic thresholds.

Further studies combining detailed visual field analysis, OCT, and longitudinal neuroimaging are needed to determine the clinical relevance of these findings.

### 4.2. Study Innovations

This study provides longitudinal data on visual function and retinal structure in patients with pituitary microadenomas, who have rarely been evaluated in previous ophthalmological studies. Unlike most earlier studies focusing on macroadenomas or chiasmal compression, our study included conservatively managed patients and an age- and sex-matched healthy control group. The same examination protocol was performed at baseline and after 12 months and included static and kinetic perimetry together with GC-IPL, RNFL, and central macular thickness measurements. This allowed functional changes to be assessed alongside structural OCT findings. The results provide preliminary evidence that subtle perimetric differences may occur in patients with microadenomas despite preserved visual acuity and stable OCT measurements.

## 5. Conclusions

Patients with conservatively managed pituitary microadenomas showed no consistent deterioration in visual function or retinal structure during the 12-month follow-up. BCVA, VFI, GC-IPL, RNFL, and central macular thickness remained stable. However, MD was consistently lower in the microadenoma group than in healthy controls, with large effect sizes, and PSD showed a modest increase over time, with medium effect sizes in both eyes. These findings document perimetric differences alongside stable OCT measurements, while their clinical significance remain to be established.

This prospective controlled study contributes to an insufficiently explored topic by documenting the longitudinal course of visual function and retinal structure in patients with conservatively managed pituitary microadenomas. These exploratory findings may provide a basis for future confirmatory studies involving larger cohorts, longer follow-up, and combined perimetric, OCT, and neuroimaging assessments to determine whether these changes persist or progress.

## Figures and Tables

**Table 1:** Demographic and baseline clinical characteristics.

Characteristic	Pituitary Microadenoma Group (*n* = 23)	Healthy Control Group (*n* = 23)	*p*-Value
Age, years	40.43 ± 10.12	40.35 ± 11.16	0.978
Female/male, n	21/2	21/2	1
RE BCVA, logMAR	0.00 (0.00–0.00)	0.00 (0.00–0.00)	1
LE BCVA, logMAR	0.00 (0.00–0.00)	0.00 (0.00–0.00)	1
RE IOP, mmHg	15.13 ± 2.38	16.22 ± 2.24	0.117
LE IOP, mmHg	15.39 ± 1.88	16.09 ± 1.90	0.219

Legend: Data are presented as mean ± standard deviation, median (interquartile range), or number, as appropriate. BCVA—best-corrected visual acuity; IOP—intraocular pressure; RE—right eye; LE—left eye; logMAR—logarithm of the minimum angle of resolution.

## Data Availability

The data presented in this study are available on request from the corresponding author.

## Supplementary Materials

The following supporting information can be downloaded at: https://www.mdpi.com/article/10.3390/cancers18213563/s1, Table S1: MRI-based tumor dimensions in individual patients with clinically nonfunctioning pituitary microadenomas; Table S2: Kinetic perimetry measurements at baseline and after 12 months in the microadenoma and control groups; Table S3: Average and sectoral ganglion cell complex thickness at baseline and 12-month follow-up; Table S4: Average and sectoral peripapillary retinal nerve fiber layer thickness at baseline and 12-month follow-up; Table S5: Macular thickness at baseline and 12-month follow-up.

## Abbreviations

The following abbreviations are used in this manuscript:

AUC	Area under the receiver operating characteristic curve
BCVA	Best-corrected visual acuity
GC-IPL	Ganglion cell–inner plexiform layer
GCC	Ganglion cell complex
IOP	Intraocular pressure
IQR	Interquartile range
LE	Left eye
logMAR	Logarithm of the minimum angle of resolution
MD	Mean deviation
OCT	Optical coherence tomography
PitNET	Pituitary neuroendocrine tumour
PSD	Pattern standard deviation
RE	Right eye
RNFL	Retinal nerve fibre layer
ROC	Receiver operating characteristic
SD	Standard deviation
SITA	Swedish Interactive Threshold Algorithm
VF	Visual field
VFI	Visual field index
