# Supplementary material for: Longitudinal Changes in Visual Field and Optical Coherence Tomography Parameters in Patients with Pituitary Microadenomas: A 12-Month Controlled Study
Source: Cancers (Basel). 2026 Nov 6;18(21):3563. doi: 10.3390/cancers18213563 (PMC13761700; doi:10.3390/cancers18213563)
Supplement: Supplementary file 1 [file cancers-18-03563-s001.zip › cancers-4614086-supplementary.pdf]

Supplementary Table S1. MRI-based tumor dimensions in individual patients with clinically nonfunctioning pituitary microadenomas.

**Patient number**	**Tumor size (mm)**		
	**Dimension 1**	**Dimension 2**	**Dimension 3**
1.	3.5	2	3
2.	4	3	3
3.	9	5	5
4.	4	4	3
5.	8	7	9
6.	6	5	6
7.	4	6	6
8.	2	2	3
9.	4	3	2
10.	6	5	7
11.	7	5	5.5
12.	3.5	3	3.3
13.	5	5	4
14.	5	3	5
15.	3	3	4
16.	2	1.5	3
17.	2	3	2
18.	7	7	6
19.	7	5	9
20.	3.5	3.5	4
21.	9	6	5
22.	4	3	3
23.	6	5	4

Table legend: Tumor dimensions are reported in millimeters. MRI, magnetic resonance imaging.

Supplementary Table S2. Kinetic perimetry measurements at baseline and after 12 months in the microadenoma and control groups

**Meridian**	**Eye**	**Group**	**Baseline, median [Q1–Q3]**	**12 months, median [Q1–Q3]**	**Within-group p**	**Between-group p: baseline / 12 months**
Superior	Right	Microadenoma	50 [50–50]	50 [45–50]	0.767	0.098 / 0.0047
		Control	50 [45–50]	50 [45–50]	0.142	
	Left	Microadenoma	50 [50–50]	50 [45–50]	0.331	0.077 / 0.028
		Control	50 [45–50]	50 [45–50]	0.021	
Nasal	Right	Microadenoma	50 [50–50]	50 [50–55]	0.789	0.178 / 0.387
		Control	50 [50–50]	50 [50–55]	0.686	
	Left	Microadenoma	50 [50–55]	50 [50–55]	0.043	0.880 / 0.472
		Control	50 [50–55]	50 [50–55]	0.075	
Inferior	Right	Microadenoma	65 [65–65]	65 [65–65]	>0.999	0.983 / 0.588
		Control	65 [65–65]	65 [65–65]	0.398	
	Left	Microadenoma	65 [65–65]	65 [65–65]	0.423	0.228 / 0.677
		Control	65 [65–65]	65 [65–65]	0.753	
Temporal	Right	Microadenoma	85 [80–90]	85 [80–90]	0.234	0.0075 / 0.0005
		Control	90 [85–90]	90 [80–90]	0.715	
	Left	Microadenoma	85 [80–90]	85 [80–90]	0.418	0.486 / 0.305
		Control	85 [80–90]	85 [80–90]	0.551	

Legend: Data are presented as median [interquartile range, Q1–Q3] and expressed in degrees. Q1, first quartile; Q3, third quartile. Within-group *p*-values refer to comparisons between baseline and 12-month measurements using the Wilcoxon signed-rank test. Between-group *p*-values refer to comparisons between the microadenoma and control groups using the Mann–Whitney U test at baseline and 12 months, respectively.

Supplementary Table S3. Average and sectoral ganglion cell complex thickness at baseline and 12-month follow-up

GC-IPL parameter	Eye	Group	Baseline, µm	12 months, µm	Within-group p	Between-group p: baseline / 12 months
Average	Right	Microadenoma	81 [77-85]	81 [77-85]	0.5337	0.7619 / 0.8962
		Control	81 [78-85]	81 [78-83]	0.5862	
	Left	Microadenoma	81 [78-84]	81 [78-84]	0.5751	0.7048 / 0.4358
		Control	79 [78-84]	80 [77-84]	0.4631	
Superior	Right	Microadenoma	80 [78-85]	80 [77-86]	0.2868	0.8977 / 0.9070
		Control	83 [78-88]	83 [79-88]	0.3144	
	Left	Microadenoma	81.5 [78-84]	81 [77-85]	0.9434	0.5544 / 0.4517
		Control	79 [78-86]	80 [78-86]	0.7413	
Superior-nasal	Right	Microadenoma	82 [79-85]	83 [79-86]	0.6247	0.8171 / 0.9149
		Control	82 [80-87]	84 [79-86]	0.1023	
	Left	Microadenoma	82 [80-86]	83 [80-86]	0.8971	0.3177 / 0.2831
		Control	83 [76-86]	82 [78-87]	0.7210	
Inferior-nasal	Right	Microadenoma	80 [78-84]	82 [78-85]	0.2343	0.5016 / 0.5158
		Control	82 [78-83]	81 [79-84]	0.4326	
	Left	Microadenoma	82 [78-84]	82 [78-85]	0.3627	0.4033 / 0.6913
		Control	81 [77-84]	80 [77-86]	0.1305	
Inferior	Right	Microadenoma	80.65 ± 6.16	80.45 ± 6.55	0.3815	0.5382 / 0.4398
		Control	79.65 ± 4.67	79.13 ± 4.73	0.1619	
	Left	Microadenoma	79 [75-83]	79 [75-84]	0.8203	0.5141 / 0.5496
		Control	78 [77-82]	79 [77-83]	0.5566	
Inferior-temporal	Right	Microadenoma	82.00 ± 6.09	81.87 ± 5.96	0.4791	0.7608 / 0.7676
		Control	81.52 ± 4.36	81.91 ± 2.99	0.8030	
	Left	Microadenoma	81.23 ± 6.03	81.64 ± 6.16	0.7603	0.8006 / 0.5282
		Control	80.83 ± 4.48	80.61 ± 4.60	0.4961	

Superior-temporal	Right	Microadenoma	80 [75-84]	80 [74-85]	0.3329	0.9074 / 0.6092
		Control	80 [76-83]	80 [76-82]	0.0442	
	Left	Microadenoma	80.5 [76-83]	80 [77-84]	0.8261	0.9485 / 0.5994
		Control	80 [77-83]	79 [77-83]	0.2790	

Legend: Data are presented as median [interquartile range, Q1-Q3. GCC, ganglion cell complex; Q1, first quartile; Q3, third quartile. Between-group comparisons were performed using the Mann–Whitney U test. The two between-group p-values refer to baseline and 12 months, respectively. Bold indicates nominal statistical significance ($p < 0.05$).

Supplementary Table S4. Average and sectoral peripapillary retinal nerve fiber layer thickness at baseline and 12-month follow-up

RNFL parameter	Eye	Group	Baseline, µm	12 months, µm	Within-group p	Between-group p: baseline / 12 months
Average	Right	Microadenoma	93 [87-102]	94 [87-101]	0.3981	0.8172 / 0.4543
		Control	93 [89-96]	93 [89-96]	0.4446	
	Left	Microadenoma	91 [86-97]	93 [87-99]	0.2659	0.8085 / 0.8000
		Control	92 [90-94]	92 [90-94]	0.3942	
Superior	Right	Microadenoma	115 [104-126]	117 [112-122]	0.1396	0.1946 / **0.0203**
		Control	111 [105-117]	110 [104-119]	0.0514	
	Left	Microadenoma	119.57 ± 13.27	120.61 ± 13.57	0.4058	0.0555 / **0.0388**
		Control	112.35 ± 8.54	112.65 ± 8.55	0.5399	
Nasal	Right	Microadenoma	70 [61-79]	71 [61-80]	0.5595	0.7682 / 0.8602
		Control	71 [66-79]	71 [64-79]	0.1913	
	Left	Microadenoma	69 [60-76]	71 [60-76]	0.7022	0.3096 / 0.3249
		Control	71 [65-79]	70 [65-79]	0.5901	

Inferior	Right	Microadenoma	125 [112-132]	128 [112-132]	0.2860	0.8950 / 0.5973
		Control	121 [113-129]	122 [115-130]	0.9579	
	Left	Microadenoma	121 [108-134]	124 [113-135]	0.0793	0.9387 / 0.8259
		Control	124 [115-127]	124 [117-129]	0.0702	
Temporal	Right	Microadenoma	66.17 ± 9.41	68.52 ± 9.83	0.1024	0.4626 / 0.9303
		Control	68.35 ± 10.46	68.78 ± 10.28	0.1909	
	Left	Microadenoma	64.04 ± 9.81	63.65 ± 8.50	0.9058	0.9562 / 0.9318
		Control	63.87 ± 11.49	63.39 ± 11.80	0.2352	

Legend: Data are presented as median [interquartile range, Q1-Q3]. RNFL, retinal nerve fiber layer; Q1, first quartile; Q3, third quartile. Between-group comparisons were performed using the Mann–Whitney U test. The two between-group p-values refer to baseline and 12 months, respectively. Bold indicates nominal statistical significance ($p < 0.05$).

Supplementary Table S5. Macular thickness at baseline and 12-month follow-up

**Parameter**	**Eye**	**Group**	**Baseline, μm**	**12 months, μm**	**Within-group p**	**Between-group p: baseline / 12 months**
Macular thickness	Right	Microadenoma	254 [240-264]	251 [240-265]	0.8329	0.4606 / 0.5673
		Control	247 [242-256]	248 [241-256]	0.8261	
	Left	Microadenoma	250 [242-263]	251 [238-266]	0.3135	0.5524 / 0.6443
		Control	250 [242-256]	250 [242-256]	0.5732	

Legend: Data are presented as median [Q1-Q3]. Q1, first quartile; Q3, third quartile. Between-group comparisons were performed using the Mann-Whitney U test. The two between-group p-values refer to baseline and 12 months, respectively.
